# Coronavirus disease 19 and risk of hyperglycemia among Iraqi patients

**DOI:** 10.1186/s43042-021-00207-3

**Published:** 2021-11-26

**Authors:** Ali H. Ad’hiah, Noor T. Al-Bayatee, Aeshah A. Ahmed

**Affiliations:** 1 Tropical-Biological Research Unit, College of Science, University of Baghdad, Al-Jadriya, Baghdad, Iraq; 2 Biotechnology Department, College of Science, University of Baghdad, Baghdad, Iraq

**Keywords:** COVID-19, Diabetes, Hyperglycemia, Prediabetes, Random blood glucose

## Abstract

A cross-sectional observational study was conducted on 213 patients with COVID-19 who did not have a clinical history of diabetes at hospital admission. One week after hospitalization, they were stratified by random blood glucose levels. It was found that 25.4, 22.5 and 52.1% of COVID-19 patients were classified as normoglycemia, prediabetes and diabetes, respectively. The study indicated that diabetes may be a risk factor for COVID-19 or the disease may be associated with an increased risk of developing diabetes.

The World Health Organization (WHO) and health authorities have devoted significant attention to understanding risk factors for coronavirus disease 19 (COVID-19) and their role in disease outcomes [1]. Several predictors were identified by a comparative analysis of risk factors across 17 studies, and age and diabetes were considered to be highly consistent [2]. Additional data support the concept that diabetes and other related diseases (hypertension and cardiovascular diseases) are the most common comorbidities in COVID-19 patients [3, 4]. It has also been proposed that COVID-19 is likely associated with an increased risk of developing diabetes [5]. This motivated us to conduct a study to explore the incidence of diabetes in hospitalized COVID-19 cases.

A multicenter cross-sectional observational study was conducted on 213 patients diagnosed with COVID-19 at referral hospitals for the management of COVID-19 in Baghdad during the period from 1 October to 15 November 2020. COVID-19 was molecularly diagnosed by detection of virus RNA in patients' nasopharyngeal secretions (reverse transcription–polymerase chain reaction test). The included patients were those who did not have a clinical history of diabetes when they were admitted to hospitals. One week after hospitalization, the records of these patients were inspected for age, gender, body mass index (BMI), ABO and Rh blood groups, hemoglobin (Hb), platelet count, white blood cell count (WBC), erythrocyte sedimentation rate (ESR), random blood glucose (RBG), and serum levels of alanine aminotransferase (ALT), aspartate aminotransferase (AST), alkaline phosphatase (ALP), total cholesterol, triglycerides, creatinine and blood urea nitrogen.

Continuous variables were given as mean and standard deviation (SD), and one-way analysis of variance (ANOVA) followed by Duncan's multiple-range post hoc test was used to assess significant differences. Categorical variables were expressed by number and percentage, and Pearson Chi-squared test was used to assess significant differences. A probability value (*p*) ≤ 0.05 was considered significant. The IBM SPSS Statistics 25.0 (Armonk, NY: IBM Corp.) was used to perform these analyses.

The mean age of COVID-19 patients was 57.4 ± 15.3 years. Males outnumbered females (86.4 *vs*. 13.6%). Most patients were overweight/obese (79.8%). Blood group O was the most common (37.6%), followed by blood groups A, B and AB (31.5, 21.6 and 9.4%, respectively). Rh-negative phenotype was present in 16.9% of patients. Other laboratory data were either within the normal range (Hb, platelets, ALT, AST and ALP) or above (WBC, ESR, and RBG). The RBG was higher than the normal range (269.5 ± 179.4 vs. 79 to < 140 mg/dL). Thus, hyperglycemia might have been identified in COVID-19 patients. This finding prompted us to re-examine the RBG and categorize patients into normoglycemia (79 to  < 140 mg/dL), prediabetes (140–199 mg/dL) and diabetes (≥ 200 mg/dL) according to the American Diabetes Association criteria [6]. It was found that 25.4% of COVID-19 patients were classified as normoglycemia, 22.5% as prediabetes and 52.1% as diabetes. It was also noted that the mean age paralleled the categorization gradually; 53.2 ± 16.8 years for normoglycemia, 55.6 ± 15.7 years for prediabetes and 60.4 ± 13.7 years for diabetes, and the difference was significant (*p* value = 0.01). As for gender, BMI and blood groups, their distribution in the three groups of patients did not show significant differences. The blood parameters also showed no significant differences between normoglycemia, prediabetes and diabetes. Platelets, RBG and ALP were exceptions and showed significant differences between the three groups of patients (Table 1 and Fig. 1).

**Table 1:** Characteristics of COVID-19 patients

Characteristic	All patients *N* = 213	Normoglycemia *N* = 54	Prediabetes *N* = 48	Diabetes *N* = 111	*p* value
Age; year	57.4 ± 15.3	53.2 ± 16.8^B^	55.6 ± 15.7^AB^	60.4 ± 13.7^A^	**0.01**
Gender					
Male	184 (86.4)	47 (87.0)	43 (89.6)	94 (84.7)	0.701
Female	29 (13.6)	7 (13.0)	5 (10.4)	17 (15.3)	
BMI					
Normal-weight	43 (20.2)	12 (22.2)	7 (14.6)	24 (21.6)	0.544
Overweight/obese	170 (79.8)	42 (77.8)	41 (85.4)	87 (78.4)	
Blood group					
O	80 (37.6)	23 (42.6)	15 (31.3)	43 (38.7)	0.587
A	67 (31.5)	15 (27.8)	15 (31.3)	36 (32.4)	
B	46 (21.6)	12 (22.2)	10 (20.8)	24 (21.6)	
AB	20 (9.4)	4 (7.4)	8 (16.7)	8 (7.2)	
Rh					
Negative	36 (16.9)	10 (18.5)	6 (12.5)	20 (18.0)	0.650
Positive	177 (83.1)	44 (81.5)	42 (87.5)	91 (82.0)	
Hb	14.2 ± 8.0	15.0 ± 13.5	13.6 ± 2.2	13.9 ± 2.8	0.638
PLT	271.3 ± 133.2	243.6 ± 100.4^B^	314.6 ± 131.6^A^	262.2 ± 152.1^AB^	**0.026**
WBC	13.9 ± 7.3	13.3 ± 6.8	15.5 ± 9.8	13.4 ± 6.1	0.190
ESR	58.8 ± 29.1	55.3 ± 30.5	60.5 ± 29.0	60.5 ± 28.2	0.562
RBG	269.5 ± 179.4	109.1 ± 23.7^C^	167.6 ± 19.0^B^	391.6 ± 170.6^A^	** < 0.001**
ALT	54.9 ± 47.8	44.9 ± 37.1	64.5 ± 61.0	52.9 ± 37.1	0.103
AST	41.9 ± 22.0	40.7 ± 20.2	43.4 ± 19.2	41.0 ± 25.2	0.799
ALP	98.8 ± 70.9	90.4 ± 39.6^AB^	84.3 ± 59.4^B^	116.1 ± 92.8^A^	**0.041**
CHOL	181.2 ± 87.2	165.5 ± 61.0	180.0 ± 93.4	192.9 ± 100.1	0.243
TRIG	200.3 ± 118.9	189.7 ± 119.9	211.2 ± 119.7	198.6 ± 118.3	0.676
SCr	1.7 ± 5.4	1.7 ± 5.5	1.1 ± 0.7	2.0 ± 7.0	0.670
BUN	63.7 ± 66.3	54.0 ± 31.9	59.1 ± 42.8	75.1 ± 94.9	0.205

Values are given as either mean ± standard deviation or a number followed by a percentage in parentheses

Rh: Rhesus blood group; Hb: Hemoglobin (g/dL); PLT: Platelets (10^9^/L); WBC: White blood cell (10^9^/L); ESR: Erythrocyte sedimentation rate (mm/hour); RBG: Random blood glucose (mg/dL); ALT: Alanine aminotransferase (U/L); AST: Aspartate aminotransferase; ALP: Alkaline phosphatase (U/L); CHOL: Total serum cholesterol (mg/dL): TRIG: Triglycerides (mg/dL); SCr: Serum creatinine (mg/dL); BUN: Blood urea nitrogen (mg/dL); *p*: Person Chi-square test or one-way analysis of variance probability followed by Duncan multiple range test post hoc (Significant *p*-value is marked in bold). Different superscript letters indicate significant difference between means in rows (*p* ≤ 0.05), while similar letters indicate no significant difference (*p* > 0.05)

**Fig. 1:**
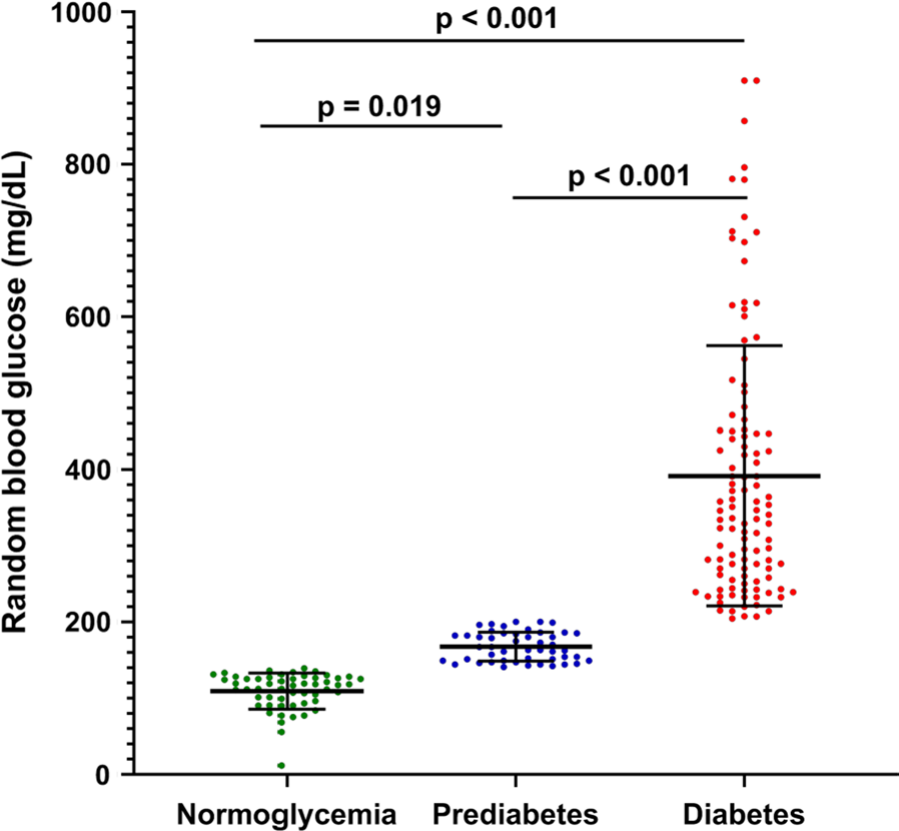
Scatter dot plot of random blood glucose in COVID-19 patients with normoglycemia (109.1 ± 23.7 mg/dL), prediabetes (167.6 ± 19.0 mg/dL) or diabetes (391.6 ± 170.6 mg/dL). Mean and standard deviations are indicated by horizontal and vertical lines respectively

The main interest of this study was to observe if COVID-19 in general is linked to an altered RBG or diabetic condition. The results indicated that this infection was associated with an increased incidence of diabetes or diabetes may be a risk factor for the development of COVID-19. Some COVID-19 patients might have developed hyperglycemia after hospitalization, while their clinical history did not indicate any evidence of diabetes; 22.5% prediabetes and 52.1% diabetes. Thus, the role of COVID-19 in the development of diabetes was proposed. Consistent with our observation, it has been reported that hyperglycemia occurred in 33.2% of COVID-19 patients during hospitalization, and the proportion was higher in severe cases compared to nonsevere cases (45.4 vs. 21.5%) [5]. Accordingly, it has been hypothesized that severe acute respiratory syndrome coronavirus 2 (SARS-CoV-2) may reduce insulin secretion through its effects on pancreatic β-cell function [7, 8]. The SARS-CoV-2 receptor (ACE2: angiotensin-converting enzyme 2) has been shown to be expressed by pancreatic cells, as well as other metabolic organs and tissues (for instance, adipose tissue, intestine and kidneys). Therefore, the virus may cause multidirectional changes in glucose metabolism that could complicate pre-existing diabetes or lead to the development of hyperglycemia in non-diabetic patients [9]. Furthermore, it has also been demonstrated that COVID-19 patients are markedly characterized by elevated serum levels of pro-inflammatory cytokines; for instance, interleukin (IL)-1, IL-6, IL-12, interferon-gamma (IFN-γ) and tumor necrosis factor-alpha (TNF-α) [10, 11]. Inflammation due to high production of cytokines may lead to insulin resistance and may impair insulin production by pancreatic β-cell [8].

Equally important, most current COVID-19 patients were overweight/obese (79.8%), and there has been strong evidence to suggest that obesity is involved in the pathogenesis of diabetes [12]. More importantly, a global estimate revealed that 45.8% of all adult diabetes cases were undiagnosed, and almost a similar proportion was reported in Iraq (47.05%) [13]. Therefore, although the patients of this study did not have a clinical history of diabetes, they may represent undiagnosed cases especially if we consider their overweight/obese status. Taken together, these data suggest that diabetes may be considered a risk factor for COVID-19, or the infection may put non-diabetic patients at risk for developing diabetes. However, additional prospective studies are warranted to understand the pathophysiology of COVID-19 in diabetic and non-diabetic patients, and to limit confounding biases due to observational design [14].

## Data Availability

The datasets used and/or analyzed during the current study are available from the corresponding author on reasonable request.

## Abbreviations

ACE2: Angiotensin-converting enzyme 2
BMI: Body mass index
COVID-19: Coronavirus disease 19
IFN-γ: Interferon-gamma
IL: Interleukin-1 (IL-1)
RBG: Random blood glucose
SARS-CoV-2: Severe acute respiratory syndrome coronavirus 2
TNF-α: Tumor necrosis factor-alpha

## References

[1] Sohrabi C, Alsafi Z, O’Neill N, Khan M, Kerwan A, Al-Jabir A et al (2020) World Health Organization declares global emergency: a review of the 2019 novel coronavirus (COVID-19). Int J Surg 76:71–76. 10.1016/j.ijsu.2020.02.034 32112977 10.1016/j.ijsu.2020.02.034 PMC7105032

[2] Rod JE, Oviedo-Trespalacios O, Cortes-Ramirez J (2020) A brief-review of the risk factors for covid-19 severity. Rev Saude Publica 54:60. 10.11606/S1518-8787.2020054002481 32491116 10.11606/s1518-8787.2020054002481 PMC7263798

[3] Zhang P, Wang M, Wang Y, Wang Y, Li T, Zeng J et al (2021) Risk factors associated with the progression of COVID-19 in elderly diabetes patients. Diabetes Res Clin Pract 171:108550. 10.1016/j.diabres.2020.108550 33232760 10.1016/j.diabres.2020.108550 PMC7833744

[4] Tadic M, Cuspidi C, Sala C (2020) COVID-19 and diabetes: is there enough evidence? J Clin Hypertens 22:943–948. 10.1111/jch.13912 10.1111/jch.13912 PMC7300807 32472662

[5] Li X, Xu S, Yu M, Wang K, Tao Y, Zhou Y et al (2020) Risk factors for severity and mortality in adult COVID-19 inpatients in Wuhan. J Allergy Clin Immunol 146:110–118. 10.1016/j.jaci.2020.04.006 32294485 10.1016/j.jaci.2020.04.006 PMC7152876

[6] Association AD (2019) Classification and diagnosis of diabetes: standards of medical care in diabetesd2019. Diabetes Care 42:S13-28. 10.2337/dc19-S002 30559228 10.2337/dc19-S002

[7] Ceriello A (2020) Hyperglycemia and COVID-19: What was known and what is really new? Diabetes Res Clin Pract 167:108383. 10.1016/j.diabres.2020.108383 32853690 10.1016/j.diabres.2020.108383 PMC7445137

[8] Ceriello A, De Nigris V, Prattichizzo F (2020) Why is hyperglycaemia worsening COVID-19 and its prognosis? Diabetes Obes Metab 22:1951–1952. 10.1111/dom.14098 32463166 10.1111/dom.14098 PMC7283840

[9] Rubino F, Amiel SA, Zimmet P, Alberti G, Bornstein S, Eckel RH et al (2020) New-onset diabetes in Covid-19. N Engl J Med 383:789–790. 10.1056/nejmc2018688 32530585 10.1056/NEJMc2018688 PMC7304415

[10] Costela-Ruiz VJ, Illescas-Montes R, Puerta-Puerta JM, Ruiz C, Melguizo-Rodríguez L (2020) SARS-CoV-2 infection: the role of cytokines in COVID-19 disease. Cytokine Growth Factor Rev 54:62–75. 10.1016/j.cytogfr.2020.06.001 32513566 10.1016/j.cytogfr.2020.06.001 PMC7265853

[11] Raony Í, Saggioro de Figueiredo C (2020) Retinal outcomes of COVID-19: Possible role of CD147 and cytokine storm in infected patients with diabetes mellitus. J Clean Prod 165:108280. 10.1016/j.diabres.2020.108280 10.1016/j.diabres.2020.108280 PMC7314672 32592839

[12] Chobot A, Górowska-Kowolik K, Sokołowska M, Jarosz-Chobot P (2018) Obesity and diabetes—not only a simple link between two epidemics. Diabetes Metab Res Rev 34:e3042. 10.1002/dmrr.3042 29931823 10.1002/dmrr.3042 PMC6220876

[13] Beagley J, Guariguata L, Weil C, Motala AA (2014) Global estimates of undiagnosed diabetes in adults. Diabetes Res Clin Pract 103:150–160. 10.1016/j.diabres.2013.11.001 24300018 10.1016/j.diabres.2013.11.001

[14] Cariou B, Pichelin M, Goronflot T, Gonfroy C, Marre M, Raffaitin-Cardin C et al (2021) Phenotypic characteristics and prognosis of newly diagnosed diabetes in hospitalized patients with COVID-19: results from the CORONADO study. Diabetes Res Clin Pract 175:108695. 10.1016/j.diabres.2021.108695 33577905 10.1016/j.diabres.2021.108695 PMC7872857

